# Pretransplant, Th17 dominant alloreactivity in highly sensitized kidney transplant candidates

**DOI:** 10.3389/frtra.2024.1336563

**Published:** 2024-04-08

**Authors:** Sarita Negi, Alissa K. Rutman, Chee Loong Saw, Steven Paraskevas, Jean Tchervenkov

**Affiliations:** ^1^ Infectious Diseases and Immunity in Global Health Program, Research Institute of the McGill University Health Centre, Montréal, QC, Canada; ^2^ Human Islet Transplantation Laboratory, McGill University Health Centre, Montréal, QC, Canada; ^3^ Department of Medicine, McGill University, Montréal, QC, Canada; ^4^ HLA Laboratory, Division of Hematology, McGill University Health Centre, Montréal, QC, Canada; ^5^ Department of Surgery, McGill University, Montréal, QC, Canada; ^6^ Division of General Surgery and Multi-Organ Transplant Program, Department of Surgery, McGill University Health Centre, Montréal, QC, Canada

**Keywords:** transplantation, highly sensitized, sensitization, Th17 cells, alloreactivity, mixed leukocyte reaction

## Abstract

**Introduction:**

Sensitization to donor human leukocyte antigen (HLA) molecules prior to transplantation is a significant risk factor for delayed access to transplantation and to long-term outcomes. Memory T cells and their cytokines play a pivotal role in shaping immune responses, thereby increasing the risk of allograft rejection among highly sensitized patients. This study aims to elucidate the precise contribution of different CD4^+^ memory T cell subsets to alloreactivity in highly sensitized (HS) kidney transplant recipients.

**Methods and results:**

Stimulation of peripheral blood mononuclear cells (PBMC) with various polyclonal stimulating agents to assess non-specific immune responses revealed that HS patients exhibit elevated immune reactivity even before kidney transplantation, compared to non-sensitized (NS) patients. HS patients' PBMC displayed higher frequencies of CD4^+^ T cells expressing IFNγ, IL4, IL6, IL17A, and TNF*α* and secreted relatively higher levels of IL17A and IL21 upon stimulation with PMA/ionomycin. Additionally, PBMC from HS patients stimulated with T cell stimulating agent phytohemagglutinin (PHA) exhibited elevated expression levels of *IFN*γ, *IL4* and, *IL21*. On the other hand, stimulation with a combination of resiquimod (R848) and IL2 for the activation of memory B cells demonstrated higher expression of *IL17A*, *TNF*α and *IL21*, as determined by quantitative real-time PCR. A mixed leukocyte reaction (MLR) assay, employing third-party donor antigen presenting cells (APCs), was implemented to evaluate the direct alloreactive response. HS patients demonstrated notably higher frequencies of CD4^+^ T cells expressing IL4, IL6 and IL17A. Interestingly, APCs expressing recall HLA antigens triggered a stronger Th17 response compared to APCs lacking recall HLA antigens in sensitized patients. Furthermore, donor APCs induced higher activation of effector memory T cells in HS patients as compared to NS patients.

**Conclusion:**

These results provide an assessment of pretransplant alloreactive T cell subsets in highly sensitized patients and emphasize the significance of Th17 cells in alloimmune responses. These findings hold promise for the development of treatment strategies tailored to sensitized kidney transplant recipients, with potential clinical implications.

## Introduction

Sensitization, characterized by the presence of circulating antibodies against human leukocyte antigens (HLA), poses a critical challenge in clinical transplantation. Approximately 30% of patients on the kidney transplant waitlist have a calculated panel reactive HLA antibody (cPRA) level exceeding 95% and are at heightened risk of decreased access to transplantation. These highly sensitized (HS) patients express high titers of multiple donor specific HLA antibodies (DSA) and face a higher probability of positive crossmatch with potential donors. This leads to prolonged transplant waiting times, an increased comorbidity burden, and a greater risk of death (1, 2). Moreover, HS patients also experience higher rates of acute and chronic rejections, as well as inferior graft and patient survival (3–6). Notably, antibody-mediated rejection (ABMR) is the primary cause of allograft loss in HS patients (7–10). Anti-HLA antibodies are considered the major drivers of ABMR and poor graft survival following kidney transplantation in HS patients (11–13).

HLA sensitization arises from exposure to epitopes on non-self HLA molecules, which can occur through pregnancies, previous transplants, or blood transfusions. For transplant candidates, sensitization tailors their immune repertoire, giving rise to alloreactive memory cells and DSA that persist in the circulation for years. Alloreactive memory can also emerge due to cross-reactive heterologous immunity after exposure to infectious agents or through homeostatic expansion following lymphopenia (14–17). Alloreactive humoral responses are regulated by antigen-specific T helper cells, underscoring the significant role of memory T cells in the alloimmune responses of HS patients. Both memory B cells and memory T cells have been linked to a higher risk of ABMR and poor graft survival after kidney transplantation (18, 19). While strategies have been developed to study and modulate memory B cells in HS patients, the role of CD4^+^ T memory cell has not been extensively explored. Murine models of transplantation have provided direct evidence of the dominant role of CD4^+^ T memory cells in the activation of donor-reactive CD8^+^ T cells and alloantibody responses (19–23), which contribute to the development of ABMR. Conventional immunosuppressants have been highly effective in controlling alloimmune responses generated from naïve T cells (24, 25); however, alloreactive memory T cells exhibit resistance to these therapies. They can cross-react with donor's HLA antigens, provoking an augmented alloimmune response with a significant impact on transplant outcomes (20, 26–29).

Assessing the pretransplant alloreactive CD4^+^ T cells repertoire could yield new insights into the underlying mechanisms contributing to ABMR and poor transplant outcomes in sensitized patients and provide valuable information for estimating the pretransplant immunological risk before kidney transplantation for HS patients. This knowledge can be particularly useful for tailoring and improving desensitization protocols. In order to characterize the pretransplant immune status of HS patients awaiting kidney transplantation, we aimed to elucidate the CD4^+^ T cells profile and the nature of alloreactive memory CD4^+^ T cells responses, and then compare them to the responses observed in non-sensitized (NS) transplant candidates.

## Materials and methods

### Human study approval

The study received approval from the Research Ethics Board of the Research Institute of the McGill University Health Centre (12-075-GEN) and was conducted in compliance with the principles outlined in the of Declaration of Helsinki. Written informed consents were obtained from all participants for sample collection and subsequent analysis. A total of 26 adult patients with end stage renal disease (ESRD), awaiting kidney transplantation at McGill University Health Centre, Quebec, Canada (MUHC) were recruited. Peripheral blood samples were collected and processed to isolate peripheral blood mononuclear cells (PBMC).

The human spleens (*n* = 10) used in this study were retrieved from deceased organ donors at the MUHC following the receipt of written informed consent for organ donation for research purposes from the next-of-kin, facilitated by coordinators from the local organ donation organization, Transplant-Québec. The donor spleens were processed to isolate spleen mononuclear cells. Table 1 provides the description of the demographic details of the donors.

**Table 1:** Splenocyte donor's characteristics.

Number of donors	10
Age, years, mean ± SEM	46 ± 5.8
Race, Caucasian	10
Sex, male/female	7/3
BMI (kg/m^2^), mean ± SEM	31 ± 2.9

BMI, body mass index.

### HLA typing and HLA antibody analysis

Molecular HLA typing (class I and class II) for all patients and donors was conducted at the Histocompatibility Laboratory at MUHC.

Patient's calculated panel reactive antibody (cPRA) and anti-HLA class I and class II antibodies' mean fluorescence intensity (MFI) data were also obtained from the Histocompatibility Laboratory at MUHC. Patients were categorized as highly sensitized (HS; cPRA >95%), moderately sensitized (MS; cPRA between 20% and 80%) or non-sensitized (NS; cPRA <20%).

### Isolation and culture of peripheral blood mononuclear cells

Whole blood samples from NS patients (*n* = 13) and HS patients (*n* = 13) were collected in heparinized tubes. Blood was diluted with equal volume of phosphate buffered saline (PBS), and PBMC were isolated by density centrifugation on Lymphocyte isolation medium (Wisent) at 1,200 g, for 20 min at room temperature (RT). The mononuclear cells layer was collected, washed, and, if necessary, erythrocytes were lysed with RBC lysis buffer (BD). PBMC were resuspended in freezing medium containing 90% FBS (Wisent) and 10% dimethylsulfoxide (DMSO) (Sigma) and cryopreserved in liquid nitrogen for later use. PBMC were cultured in RPMI-1640 supplemented with 5% human AB serum (GemCell), penicillin-streptomycin (50 IU/ml penicillin, 50 µg/ml streptomycin; Wisent), 2 mM L-glutamine (Wisent).

### Isolation of spleen mononuclear cells

Donor spleens (*n* = 10) were preserved in University of Wisconsin solution before processing. Isolation medium was prepared with RPMI-1640 (Wisent) supplemented with 1% human AB serum (GemCell). The spleen was divided into small pieces and mechanically disrupted in the presence of isolation medium. The tissue suspension was washed and filtered through a 70 µM cell strainer to remove cells from tissue fragments. Mononuclear cells were isolated from the cell suspension by density centrifugation on Lymphocyte isolation medium (Wisent) at 1,200 g, for 20 min at RT. Spleen mononuclear cells were collected, washed, filtered, and cryopreserved in freezing medium containing 90% FBS (Wisent) and 10% DMSO (Sigma).

### Flow cytometry analysis of cytokines expression

The frequency of cytokine (IFNγ, IL4, IL6, IL17A, and TNFα) expressing CD4^+^ cells was determined by flow cytometry following intracellular staining. PBMC (2 × 10^5^ cells) were cultured in a 96-well, U-bottom plate in 0.2 ml/well culture medium at 37°C, 5% CO_2_ for 3 days. Subsequently, cultures were stimulated for 4 h with a cell stimulation cocktail (CSC) containing phorbol 12-myristate 13-acetate (PMA), ionomycin, brefeldin A, and monensin (Life Technologies). Supernatants were collected and stored at −80 °C and used for measuring cytokine levels. Cells were harvested and stained with anti-CD4-EF450; anti-CD8-PE-Cy7 and anti-CD19-AF700. Cells were washed, fixed, permeabilized with IC fixation and permeabilization buffer set (Life Technologies), followed by intracellular staining with anti-IFNγ-APC-EF780, anti-IL4-APC, anti-IL6-PerCP-EF710, anti-IL17A-PE, and anti-TNFα-AF488. All reagents were from Life Technologies unless otherwise indicated.

Cells were always stained with Fixable Viability Dye (FVD) EF506 or FVD-BUV496 (Life Technologies) to exclude dead cells from analysis. Doublets were excluded using forward scatter height against forward scatter area and subsequently side scatter height against side scatter area. Fluorescent minus one controls were used for gating intracellular cytokines. All data were acquired on an LSRFortessa cytometer (BD) and data analysis was carried out with FlowJo software (Tree Star, Inc.).

### Analysis of cytokines in cell culture supernatants

To measure secreted cytokine levels, supernatants of patients' PBMC cultures activated with CSC were collected. Multiplex immunoassays were carried out using the 25 µl of neat supernatant and a 14-plex Milliplex human Th17 magnetic bead panel (EMD Millipore) according to the manufacturer's protocol. Cytokines measured were IFNγ, IL4, IL6, IL17A, IL17E, IL17F, IL21, IL10, IL13, IL22, IL23, IL31, IL33, and TNFα. Luminex Magpix plate reader (Luminex) was used, and median fluorescence intensity (MFI) data were analyzed with 5-parameter logistic to calculate the cytokine concentration in samples using Milliplex analyst software (Merck Millipore). Minimum detectable concentrations (MinDC) of cytokines were IFNγ (6.02 pg/ml), IL6 (4.32 pg/ml), IL17A (1.21 pg/ml), IL21 (2.7 pg/ml), IL22 (17.54 pg/ml), and TNFα (0.49 pg/ml). Other cytokines were below detection limit in our samples in our optimization experiments. MFI values below minDC were set to half of minDC for measured cytokines.

### IL17A enzyme-linked immunosorbent assay (ELISA)

PBMC (2 × 10^5^ cells) from NS and HS patients were stimulated with 5 μg/ml phytohemagglutinin (PHA; Sigma) for 24 h and culture supernatants were collected and stored at −80°C until use. ELISA was performed using Human IL17A ELISA kit (Life Technologies) following manufacturer's instructions.

### Real-time polymerase chain reaction (PCR)

PBMC (2 × 10^5^ cells) from NS and HS patients were stimulated with either 5 μg/ml phytohemagglutinin (PHA; Sigma) or with a combination of resiquimod (R848) (1 µg/ml; R&D Systems) and human IL-2 (10 ng/ml; R&D Systems) for 24 h. Cells were harvested and RNA was extracted using RNeasy micro-plus kit (Qiagen). cDNA was synthesized using iScript cDNA synthesis kit (Biorad). Real-time PCR amplifications were performed using Taqman Fast Advanced Master Mix (Life Technologies) and were run on MyiQ2 real-time detection system (Biorad). The following Taqman assays were used for each gene: *IFN*γ (Hs00989291_m1), *IL4* (Hs00932431_m1), *IL6* (Hs00985639_m1), *IL17A* (Hs00174383_m1), *TNF*α (Hs01113624_g1), *IL21* (Hs00222327_m1). The stability of potential reference genes *TBP* (Hs00427620_m1), *B2M* (Hs00187842_m1), *RNA18S 1* (Hs99999901_s1) and *PPIA* (Hs04194521_s1) mRNA expression levels was evaluated using RefFinder, a web-based tool which recommended the use of geometric mean of *B2M* and *TBP* as the most stable normalization factor for our samples (30). Geometric mean of housekeeping genes *TBP* and *B2M* was used for normalization and fold changes were calculated using the 2^−ΔΔCt^ (Livak) Method.

### Preparation and x-ray irradiation of donor antigen presenting cells

Spleen mononuclear cells were thawed, and donor antigen presenting cells (APCs) were isolated by depleting T cells using CD2 dynabeads (Life Technologies), following the manufacturer's instructions. T cells depleted fraction or donor APCs were suspended in culture medium RPMI-1640 supplemented with 5% human AB serum (GemCell), penicillin-streptomycin (50 IU/ml penicillin, 50 µg/ml streptomycin; Wisent), 2 mM L-glutamine (Wisent). Donor APCs were exposed to x-ray irradiation dose of 20 Gray (Gy) to inactivate cells. Faxitron x-ray machine (Faxitron x-ray Corporation, IL) was used for irradiation at a voltage of x-ray tube set to 160 kV, current of 6.3 mA and a dose rate of 0.629 Gy/min. Cells were washed with culture medium twice and used as allogeneic stimulators in mixed lymphocyte reaction.

### Allogeneic one-way mixed lymphocyte reaction (MLR) to measure cytokine expression

To assess allogeneic lymphocyte response, one-way MLR was performed. Patient's PBMC (2 × 10^5^ cells) or responders were co-cultured with irradiated donor APCs stimulators in 1:1 ratio for 6 days. On the 6th day, cultures were stimulated using CSC for 4 h, and processed for flow cytometry to measure cytokine expression. Cells were stained with FVD EF506, anti-CD4-EF450; anti-CD8-PE-Cy7 and anti-CD19-AF700. Cells were fixed and permeabilized with IC fixation and permeabilization buffer set (Life Technologies), followed by intracellular staining with anti-IFNγ-APC-EF780, anti-IL4-APC, anti-IL6-PerCP-EF710, anti-IL17A-PE, and anti-TNFα-AF488.

### Allogeneic one-way MLR to measure memory cell responses

Responder PBMC were labeled with fluorogenic dye carboxyfluorescein succinimidyl ester (CFSE) (Life Technologies) at a final concentration of 5 µM. One-way MLR was performed by priming patient PBMC (2 × 10^5^ cells) with irradiated donor APCs in 1:1 ratio for 24 h followed by activation with anti-CD3 (0.1 µg/ml) and anti-CD28 (2 µg/ml) for an additional 4 days. Cells were stained for flow cytometry with anti-CD4-EF450, anti-CD25-BV786 (BD Bioscience), anti-CD45RA-PE-Cy7, anti-CD45RO-PE-Cy5, and anti-CCR7-APC. To determine the frequencies of CD4 memory T cell subsets in response to allogeneic stimulation, CD4 cells were gated on effector memory (T_EM_, CD4^+^CCR7^−^CD45RO^+^), central memory (T_CM_, CD4^+^CCR7^+^CD45RO^+^), naïve (T_N_, CD4^+^CCR7^+^CD45RO^−^), and terminally differentiated effector (T_EMRA_ CD4^+^CCR7^−^CD45RO^−^). The proliferation of allo-activated T cell subsets was determined by quantifying CFSE dilution. FlowJo proliferation analysis platform was used to calculate proliferation index. Activation of indicated T cell subsets was measured by CD25 expression.

### Statistical analysis

Statistical analysis was performed using GraphPad Prism software. Categorical variables are expressed as the corresponding number (*n*). Categorical variables between HS and NS patients were compared using the Fisher's exact test. Continuous variables are expressed as mean ± standard error of the mean (SEM). Data were analyzed using unpaired, two-tailed Mann-Whitney test. *P*-values less than 0.05 were considered statistically significant.

## Results

### Patient's characteristics

In this study, a total of 13 NS and 13 HS patients were recruited. A comparison of clinical characteristics between NS and HS patients is presented in Table 2. No significant differences were observed in terms of age, sex, body mass index (BMI), type of dialysis and cause of ESRD between the two groups. All NS patients have cPRA below 20%; all HS patients have cPRA greater than 95%. HS patients had longer duration on dialysis with a mean time of 3.8 ± 0.8 years for NS patients and 12.3 ± 1.4 years for HS patients. Only one patient in NS group and eight patients in HS group had received a previous kidney transplant. In the NS group, twelve patients have undergone kidney transplantation as compared to two patients in the HS group within the 5-year period following recruitment.

**Table 2:** Patient's characteristics.

Parameter	NS (*n* = 13)	HS (*n* = 13)	*P* value
Age, years, mean ± SEM	56.7 ± 3.2	52.3 ± 2.5	0.35
cPRA (>95%)	0	13	0.0001*
Sex, male/female	8/5	7/6	1.0
BMI (kg/m^2^), mean ± SEM	28.4 ± 1.5	27.7 ± 1.4	0.77
Dialysis type, HD	9	11	0.65
Time on dialysis, years, mean ± SEM	3.6 ± 0.8	12.3 ± 1.4	<0.0001*
Previous transplant	1	8	0.01*
Cause of ESRD			
Diabetes	7	3	0.2
Glomerulosclerosis	1	4	0.3
Systemic lupus erythematosus	0	1	1.0
IgA nephropathy	0	3	0.2
Polycystic kidney	2	0	0.5
Hypertension	2	1	1.0
Other	1	1	1.0
Transplanted	12	3	0.003*

NS, non-sensitized; HS, highly sensitized; cPRA, calculated panel reactive HLA antibody; BMI, body mass index; HD, hemodialysis; ESRD, end stage renal disease.

* *p*-value < 0.05.

### PBMC from HS patients express higher levels of cytokines

**Figure 1:**
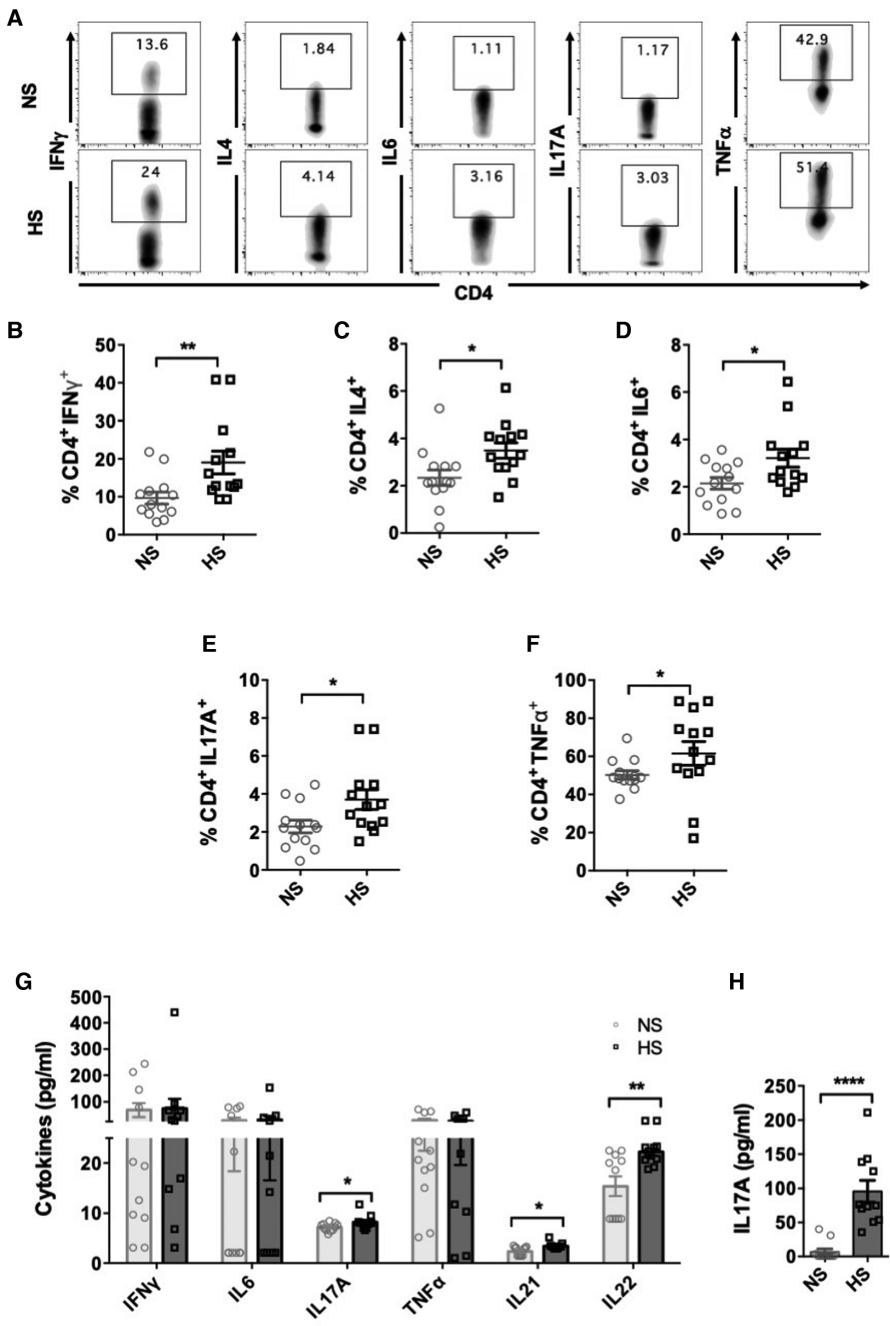
CD4^+^ T cells cytokine expression in HS and NS patients. PBMC from HS (*n* = 13) or NS (*n* = 13) patients were cultured for 3 days and stimulated with PMA/ionomycin for 4 h followed by flow cytometry assay. (**A**) Representative flow plots showing cytokine expressing (IFNγ, IL4, IL17A, IL6, TNFα) CD4^+^ T cells in NS and HS patients. Scatter plots comparing percentages of CD4^+^ T cells expressing (**B**) IFNγ, (**C**) IL4, (**D**) IL6, (**E**) IL17A, and (**F**) TNFα in HS and NS patients. (**G**) Measurements of cytokine levels released in culture supernatants using Luminex assays. (**H**) PBMC from NS (*n* = 11) and HS patients (*n* = 11) were stimulated with PHA for 24 h and levels of IL17A secreted in culture supernatants was measured by ELISA. Data shows mean ± SEM (*p < 0.05, **p < 0.005, ***p < 0.0005, ****p < 0.00005).

To investigate and compare the functional profile of CD4^+^ T cells between NS and HS patients, we analyzed the intracellular cytokine expression of CD4^+^ T cells. PMA/ionomycin is known to be suitable for rapid and effective stimulation of T cells for short time frames. They activate several intracellular signaling pathways, resulting in T cell activation and production of several cytokines (31). Therefore, we utilized PMA/ionomycin for stimulation and PBMC from both NS or HS patients were cultured for 3 days and stimulated with CSC for 4 h, followed by flow cytometry analysis. HS patients exhibited significantly higher percentages of CD4^+^ T cells expressing IFNγ, IL4, IL6, IL17A and TNFα as compared to NS patients and (Figures 1A–F).

We further assessed the levels of cytokine release by PBMC from HS and NS patients by measuring cytokine levels in the supernatants of stimulated PBMC cultures. PBMC from HS patients secreted significantly higher levels of IL17A as compared to NS patients (Figure 1G). There was no significant difference in the production of IFNγ, IL6 and TNFα in the supernatants between HS and NS patients. The levels of IL4 were below the detection limit and therefore could not be assessed. Th17 cells are also known to produce IL21 and IL22, and we observed that levels of both IL21 and IL22 were elevated in the supernatants of HS patients' PBMC cultures (Figure 1G). We also stimulated patients' PBMC with PHA and measured levels of IL17A in the culture supernatants. Significantly higher levels of IL17A were observed in HS patients' PBMC cultures (Figure 1H). We speculate that variations in cytokine expression detected through flow cytometry, Luminex, or ELISA may stem from various technical factors including cytokine stability, freeze-thaw cycles, and sensitivity of the commercial kit employed. In summary, our results demonstrated that under inflammatory conditions, PBMC from HS patients expressed higher levels of cytokines associated with Th1, Th2 and Th17 cells as compared to NS patients.

### Alterations in the cytokine mRNA profile of PBMC from HS and NS patients

**Figure 2:**
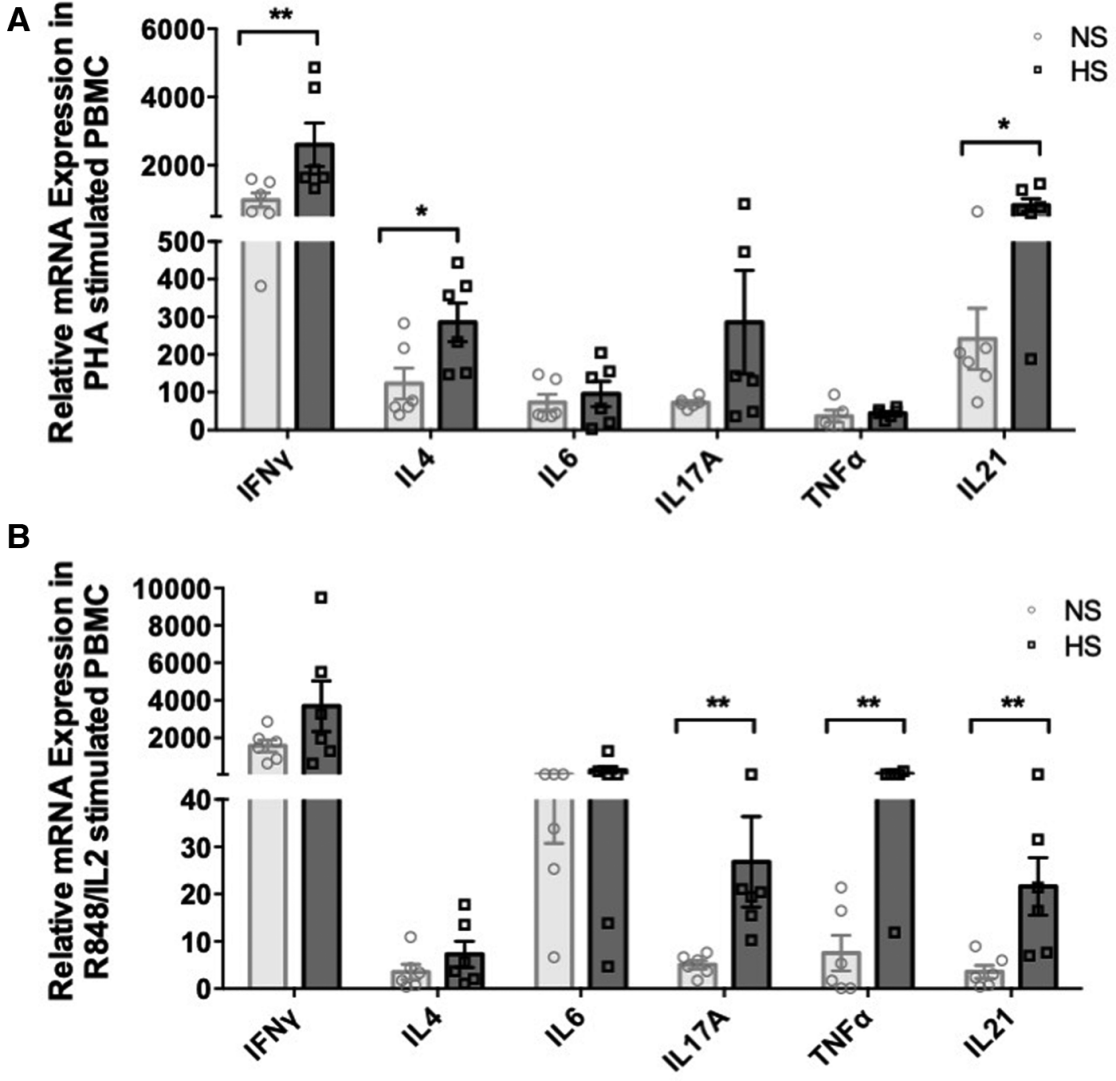
Cytokine mRNA expression in PBMC from HS and NS patients. PBMC from HS (*n* = 6) or NS (*n* = 6) patients were stimulated with (**A**) PHA or (**B**) R848/IL2 for 24 h. The mRNA expression for *IFN*γ, *IL4*, *IL6*, *IL17A*, *TNF*α, and *IL21* was measured by real-time PCR. The data represent mean ± SEM (*p < 0.05, **p < 0.005, ***p < 0.0005, ****p < 0.00005).

We examined how different inflammatory conditions might influence the gene expression of various cytokines in PBMC from NS and HS patients and sought to compare responses generated by T and B cells activating agents. We stimulated PBMC with a combination of R848 [agonist of toll-like receptors (TLR) 7 and 8] and IL2, known to induce memory B cells proliferation and immunoglobulin secretion, for 24 h (32). PHA is another robust T-cell activating agent and stimulates expression of a wide range of cytokines than PMA/ionomycin (33). We chose to treat PBMC with PHA for 24 h as a comparable strategy for T cell activation. The PBMC were stimulated with a R848/IL2 or PHA for 24 h and real-time PCR was used to measure cytokine transcription patterns. The results indicated that PHA stimulation induced significantly higher expression of IFNγ, IL4, and IL21 genes in HS patients as compared to NS patients (Figure 2A). Whereas, stimulating PBMC with R848/IL2 led to significantly higher expression of IL17A, TNFα, and IL21 genes in HS patients (Figure 2B). These results suggest that different stimulatory environment may lead to distinct immune responses in transplant patients, and HS patients exhibit relatively stronger cytokine responses than NS patients.

### Allogeneic stimulation induces IL17A expressing CD4^+^ population

**Figure 3:**
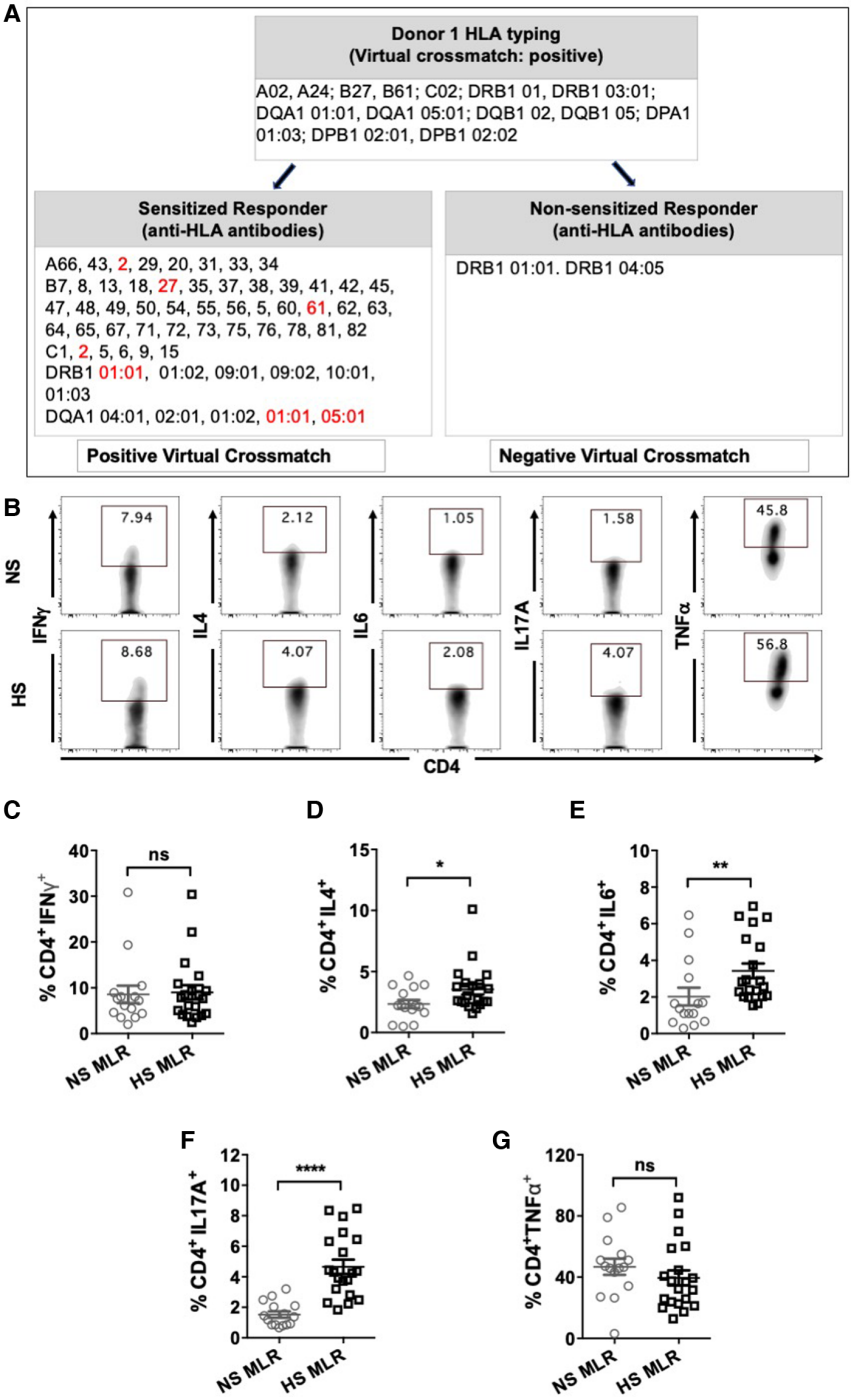
CD4^+^ T cells cytokine expression after allogeneic stimulation using MLR assay. HS (*n* = 8) or NS (*n* = 9) PBMC were co-cultured with donor APC for 6 days followed by stimulation with PMA/ionomycin and transport block for 4 h and flow cytometry assay. A total 20 HS and 15 NS responder-stimulator pairs were used. (**A**) Characteristics of a representative responder-stimulator pair form NS and HS patient used for allogeneic stimulation in MLR. Responder box shows anti-HLA antibodies detected in the patient's serum. Donor box shows donor HLA. DSA to donor HLA antigens are marked in red. (**B**) Representative flow plots showing detection of cytokine expressing (IFNγ, IL4, IL6, IL17A, TNFα) CD4^+^ T cells in NS and HS patients. Scatter plots comparing percentages of CD4^+^ T cells expressing (**C**) IFNγ, (**D**) IL4, (**E**) IL6, (**F**) IL17A, and (**G**) TNFα in NS and HS patients after MLR. Each symbol represents single responder-stimulator pair. Data shows mean ± SEM (*p < 0.05, **p < 0.005, ***p < 0.0005, ****p < 0.00005).

We sought to characterize the direct-pathway CD4^+^ T cells responses upon antigen-specific stimulation by performing an allogeneic MLR assay and measuring cytokine expression in CD4^+^ T cells. HLA antibody specificities of HS patients were matched with third-party donor's HLA to identify responder-stimulator pairs with positive virtual crossmatch (Figure 3A). MLR was also conducted using PBMC from NS patients with donor APCs. All NS patients showed negative virtual crossmatch with the donor's HLA. All responder-stimulator pairs had extensive HLA mismatches (approximately 6.8 ± 0.26 mismatches).

The donor APCs were irradiated to inactivate them and were used as stimulator cells in the MLR and co-cultured with PBMC for 6 days followed by stimulation with CSC and flow cytometry (Figure 3A). Allogeneic stimulation with donor APCs revealed increased percentages of CD4^+^ T cells expressing IL4, IL6 and IL17A in HS patients as compared to NS patients (Figure 3D–F). However, there was no significant difference in the percentages of CD4^+^ T cell populations expressing IFNγ, and TNFα between NS and HS patients (Figures 3C–G). These findings suggest that HS patients may exhibit alloimmune responses with a Th2 and Th17 pattern, which closely resemble the patterns observed in autoimmune conditions (34–37).

**Figure 4:**
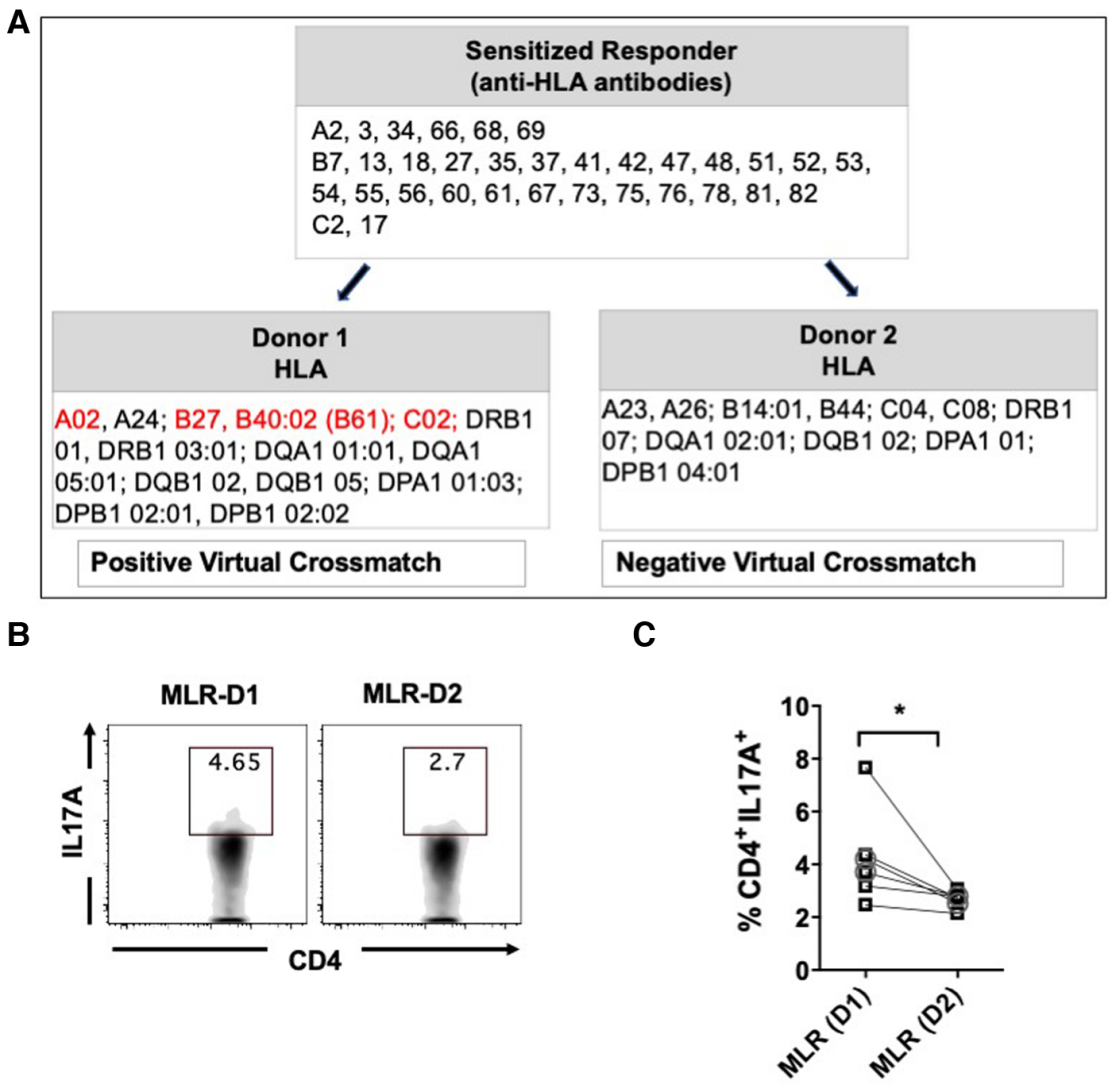
Percentages of IL17A expressing CD4^+^ T cells after MLR of PBMC from sensitized patients with donor APCs having positive (D1) or negative (D2) virtual crossmatch. Seven pairs of MLR were performed by co-culturing PBMC from HS (*n* = 5, □ symbol) or NS (*n* = 2, ○ symbol) patients with donor APCs for 6 days followed by stimulation with CSC for 4 h and flow cytometry analysis. (**A**) Characteristics of a representative responder-stimulator pair used for MLR. Recall donor HLA antigens are marked in red. (**B**) Flow plot illustrating IL17A expressing CD4^+^ T cells after stimulation with D1 or D2 donor APCs. (**C**) Scatter plot comparing the IL17A expressing CD4^+^ T cells after MLR with D1 or D2 donor APCs. The data represent mean ± SEM (*p < 0.05, **p < 0.005, ***p < 0.0005, ****p < 0.00005).

Next, we aimed to compare the CD4^+^ T cells activity of sensitized patients in response to allogeneic stimulation with donor APCs having either positive or negative virtual crossmatch. For seven sensitized patients, we identified pairs of donors with positive (D1) or negative (D2) virtual crossmatch (Figure 4A) and performed a one-way MLR followed by flow cytometry (Figure 4B). Stimulation with donor APCs that had positive virtual crossmatch induced relatively higher percentages of IL17A expressing CD4^+^ T cells as compared to stimulation with donor APC with negative crossmatch (Figure 4C). There were no significant differences in the expression of IFNγ, IL4, IL6, and TNFα expressing CD4^+^ T cells. These results indicate that Th17 associated memory pathways are predominantly induced in response to allogeneic stimulation with recall HLA antigens. This is very similar to immune patterns observed in autoimmune disease like Systemic Lupus Erythematosus (SLE) and Rheumatoid Arthritis (RA) (37).

### Allogeneic stimulation induces activation of memory CD4^+^ cells in HS patients

**Figure 5:**
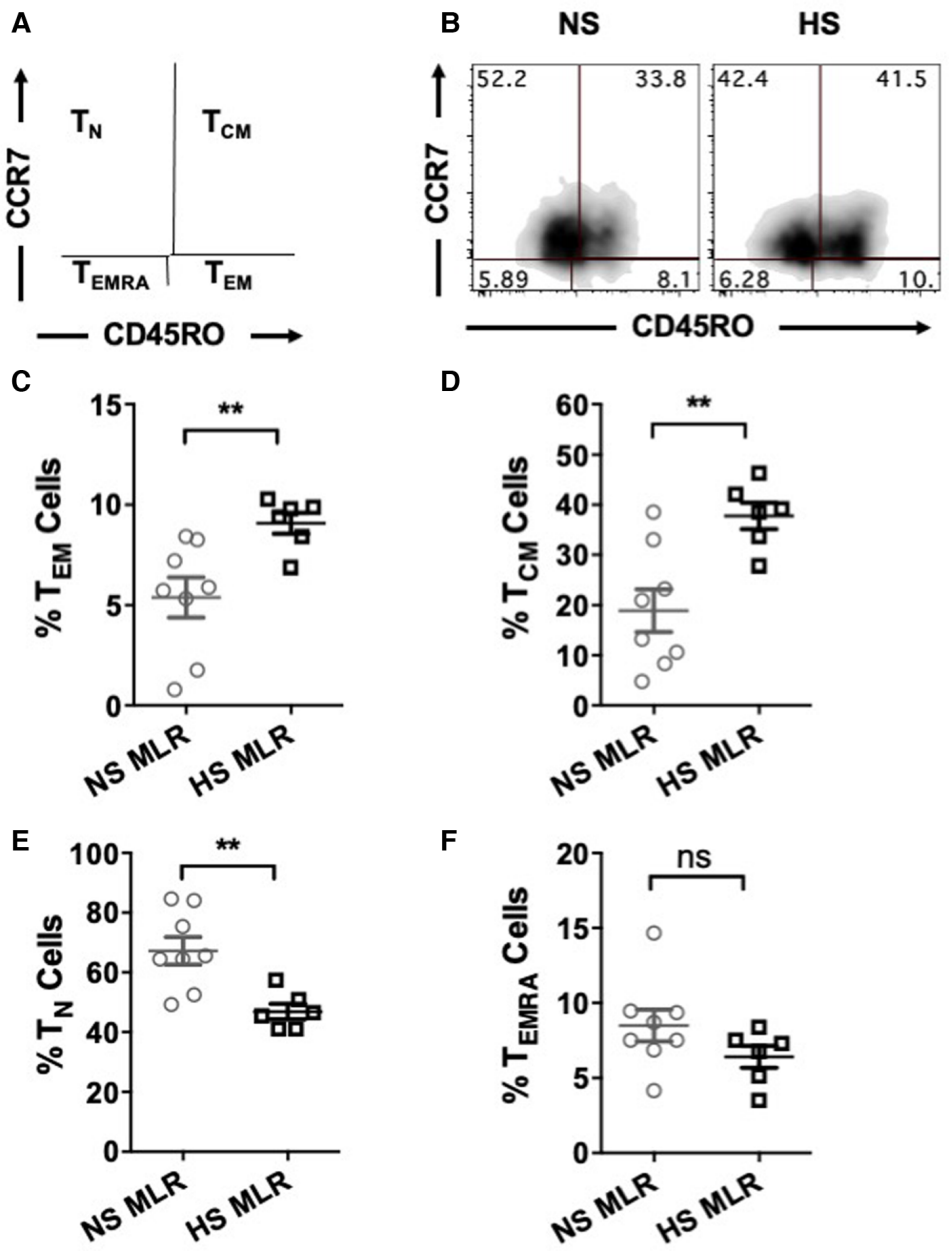
Percentages of CD4^+^ T cell subsets after allogeneic stimulation of the patient's PBMC (HS, *n* = 6; NS, *n* = 8). PBMC were primed overnight with donor APCs and subsequently stimulated with anti-CD3 and anti-CD28 for 4 more days followed by flow cytometry analysis. (**A**) Gating strategy for the memory and naïve CD4^+^ T cell subsets; T_EM_ (CD45RO^+^CCR7^−^), T_CM_ (CD45RO^+^CCR7^+^), T_N_ (CD45RO^−^CCR7^+^), and T_EMRA_ (CD45RO^−^CCR7^−^). (**B**) Representative dot plots showing percentages of T cell subsets. Scatter plot comparing percentages of CD4^+^ T cell subsets (**C**) T_EM,_ (**D**) T_CM,_ (**E**) T_N,_ and (**F**) T_EMRA_ of NS or HS patients after MLR. The data represent mean ± SEM (*p < 0.05, **p < 0.005, ***p < 0.0005, ****p < 0.00005).

**Figure 6:**
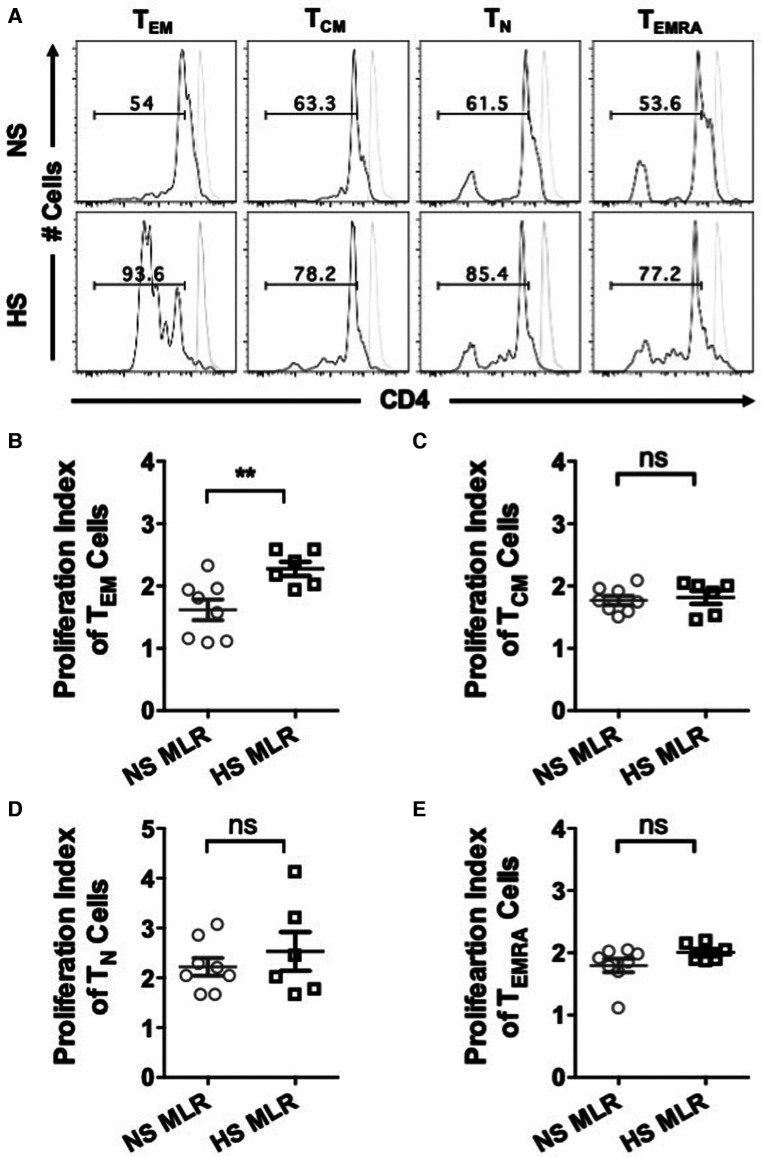
Quantifying proliferative response to allogeneic stimulation. PBMC (HS, *n* = 6; NS, *n* = 8) were primed overnight with donor APCs and subsequently stimulated with anti-CD3 and anti-CD28 for 4 more days followed by flow cytometry analysis. Proliferation of donor-reactive CD4^+^ T cell subsets were quantified using FlowJo software. (**A**) Dot plots representing proliferation in all T cell subsets of NS and HS patient groups. Scatter plot comparing proliferation indices of T cell subsets (**B**) T_EM,_ (**C**) T_CM,_ (**D**) T_N,_ and (**E**) T_EMRA_. Unstimulated cells are shown as grey histograms. The data represent mean ± SEM (*p < 0.05, **p < 0.005, ***p < 0.0005, ****p < 0.00005).

**Figure 7:**
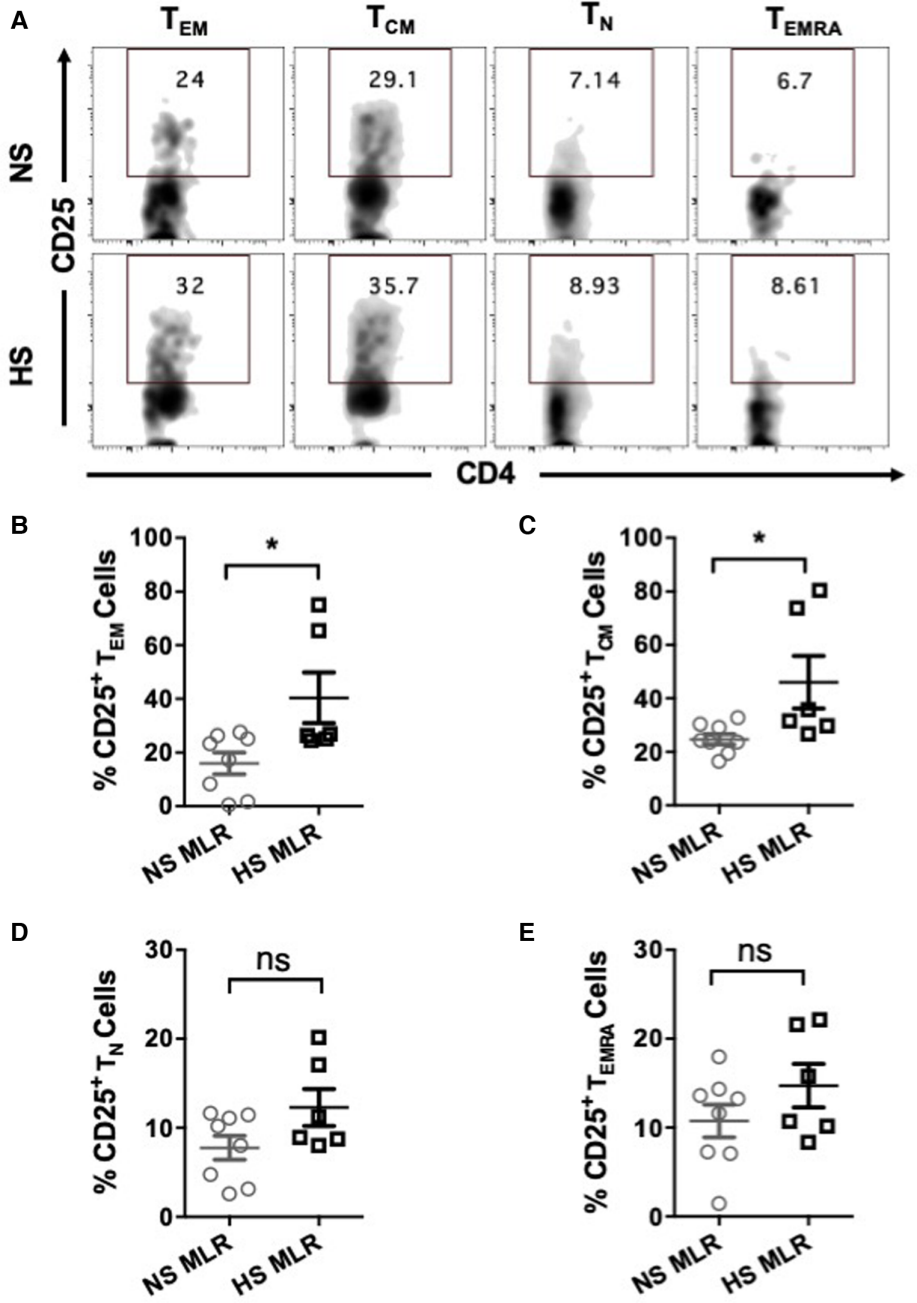
CD25 expression on CD4^+^ T cell subsets of patients’ PBMC (HS, *n* = 6; NS, *n* = 8) after allogeneic stimulation with donor APCs followed by stimulation with anti-CD3 and anti-CD28 for 4 more days and flow cytometry analysis. (**A**) The representative dot plots showing the proportion (%) of CD25 expressing cells within different T cell subsets of NS and HS patients. Scatter plot comparing percentages of CD25 expression among T cell subsets (**B**) T_EM,_ (**C**) T_CM,_ (**D**) T_N,_ and (**E**) T_EMRA_ of NS or HS patients. The data represent mean ± SEM (*p < 0.05, **p < 0.005, ***p < 0.0005, ****p < 0.00005).

We further investigated whether allogenic stimulation also affects activation of CD4^+^ T memory cells. CFSE-labeled PBMC were cultured with donor APCs and assessed for the frequency of T cell subsets, including effector memory (T_EM_, CD4^+^CCR7^−^CD45RO^+^), central memory (T_CM_, CD4^+^CCR7^+^CD45RO^+^), naïve (T_N_, CD4^+^CCR7^+^CD45RO^−^), and terminally differentiated effector (T_EMRA_ CD4^+^CCR7^−^CD45RO^−^) (Figures 5A,B). HS patients exhibited relatively higher percentages of T_EM_ and T_CM_ cells as compared to NS patients (Figures 5C,D). However, the percentage of T_N_ was reduced in HS patients and percentages of T_EMRA_ were similar between the two patient groups (Figures 5E,F). We also compared the proliferation of all the CD4^+^ T cell subsets and observed that only the T_EM_ subset exhibited significantly higher proliferation in HS patients (Figures 6A,B). There was no significant difference in proliferation of T_CM_, T_N_, and T_EMRA_ cells (Figures 6C–E). These results suggest that the lower percentages of T_N_ subset observed in HS patients may be attributed to the increased proliferation of T_EM_ cells rather than a reduction in their own frequency. Additionally, we measured the expression of CD25, an activation marker that is upregulated on CD4^+^ T memory cells upon antigen stimulation, facilitating the detection of activated memory T cells (38–40) (Figure 7A). Both classes of CD4^+^ T memory cells (T_CM_ and T_EM_) showed higher expression of CD25 (Figures 7B,C). These results suggest that exposure to recall HLA antigens from donor cells stimulates a greater T_EM_ and T_CM_ memory cell response in HS patients.

## Discussion

Sensitization to donor-HLA molecules prior to kidney transplantation presents a substantial risk for allograft rejection and the subsequent graft loss following kidney transplantation. The intricate interplay of memory T cells and their cytokines plays a critical role in the development of alloimmune responses, increasing the risk of allograft rejection in sensitized patients. Our study results illustrate that HS patients exhibit elevated immune reactivity prior to transplantation.

The measurement of cytokine production in CD4^+^ T cells following activation with different non-specific polyclonal stimulating agents revealed that HS patients exhibit significantly stronger Th1, Th2 and Th17 mediated responses. Previous studies have reported that type 1 and type 2 immunities do not strictly define cellular and humoral responses, and both Th1 and Th17 memory CD4^+^ T cells contribute to B-cell differentiation and activation (41, 42). Luminex and QPCR analyses showed induction of inflammatory cytokines like IL4, IL6, IL17A, IL21 and IL22, which are known to regulate differentiation and function of Th17 and contribute to B cell activation, plasma blast differentiation, and antibody production (43–48). Our results are consistent with another study that reported increased expression of genes such as *IL2*, *IL4* and *IL21* in HS patients awaiting kidney transplantation (49). It's important to note that dysregulation of T cells is widely recognized to play a crucial role in the pathogenesis of several kidney diseases, with the Th17/IL17 axis commonly implicated in contributing to renal tissue damage in conditions such as SLE, IgA nephropathy, Type 2 diabetic nephropathy, hypertension (50–54). However, in the current study, patients in the NS and HS group were not matched based on ESRD, which could potentially influence their immune responses differently. Hence, these findings will require validation in a larger cohort.

MLR assays demonstrated that HS patients showed greater proportion of IL4, IL6 and IL17A expressing alloreactive CD4^+^ T cells compared to NS patients. Th17 donor-reactive responses predominate upon exposure to recall HLA antigens. While several studies have reported the correlation between pretransplant frequencies of donor reactive IFNγ expressing alloreactive cells and acute rejection, and impaired graft function in kidney transplant recipients (26, 55–58), we did not observe significant differences in alloreactive IFNγ expressing CD4^+^ T cells. This supports the notion that frequencies of IFNγ producing memory cells do not correlate with patient's sensitization and could represent cellular alloimmunity rather than humoral alloimmunity (59, 60).

The role of memory T cell subpopulations in memory responses is well established. We observed that HS patients showed a higher overall proportion of alloreactive T_EM_, and T_CM_ subsets, along with increased CD25 expression in both subsets. Furthermore, HS patients displayed a significantly higher proliferation index in T_EM_ only. Our results are consistent with earlier reports suggesting that T_EM_ possess greater alloimmune response capabilities (61, 62). Interestingly, relatively higher proportions of IL17 producing T_EM_ were observed in patients with chronic allograft dysfunction as compared to patients with long-term stable grafts (63). Patients with ESRD also showed higher frequencies of IL17 producing T_EM_ than healthy volunteers (63). It is possible that alloimmune responses in HS patients are dominated by Th2 and Th17 memory cells.

Th17 cells act as effectors of kidney injury and IL17A expression has been associated with allograft dysfunction and rejection (63–71). Moreover, IL17A expression by tubular epithelial cells in renal allografts undergoing acute ABMR has been reported (72), underscoring the existence of multiple sources of IL17A. Deteix et al. showed that Th17 lymphocytes promote tertiary lymphoid neogenesis and the organization of inflammatory effectors into ectopic germinal centers within the graft suggesting a critical role for Th17 cells in the local humoral immune response (70). In a murine model of MHC-Mismatched renal allograft transplantation, Kwan et al. demonstrated that IL17A deficiency [IL17(−/−)] significantly attenuated allograft injury and resulted in prolonged allograft survival. Furthermore, in these IL17(−/−) mice, splenocyte exhibited reduced production of IFNγ when stimulated, suggesting that type 1 immunity is also affected by IL17A deficiency (73). Additionally, recent data have also demonstrated a distinct profile of pretransplant regulatory T cells (Treg) in HS patients, featuring a prominent Th17-like Treg population (74). Previously we showed that pretransplant Treg suppressive cell function is an independent predictor of delayed and slow graft function (75, 76). Both Treg and Th17 populations exhibit a high degree of plasticity, enabling them to adapt to the changing inflammatory milieu during an immune response. There is a growing body of evidence that highlights the critical role of IL6 and IL21 signaling in regulating the balance between Th17 and Treg cells, as they promote the induction of Th17 cells while inhibiting Treg differentiation (77–81). Few studies have implicated IL6 in acute and chronic graft rejection (82). The plasticity of Treg and Th17 cells could be critical during alloimmune responses in HS patients, potentially shifting the Treg/Th17 balance toward a proinflammatory, Th17 dominated immune response after transplantation. Several studies have shown that a higher Th17/Treg ratio in allografts correlates with reduced allograft function and survival (67, 68). It is plausible that IL6 and IL17A signaling pathways operate synergistically, and a dysregulated inflammatory cytokine environment further accelerates allograft rejection response in sensitized patients.

Current approaches to improve graft and patient survival in HS patients rely on reducing DSA levels through desensitization or strategies targeting B cells or plasma cells, but these have had limited success (83). Calcineurin based immunosuppression has proven to have inadequate effect on Th17 cells (84). Considering the critical role of the IL17A and IL6 axis in allograft pathology of HS patients, a combination of therapies targeting these pathways may enhance allograft function and survival in HS patients. Clinical trials have explored the therapeutic potential of blocking IL17A or IL6 signaling pathways to alleviate allograft rejection. Strategies using mTOR-inhibitors (85–88) or metabolic regulators like vitamin D3 (89) to suppress Th17 pathways have shown some potential, however, their clinical relevance remains unclear. Data from preliminary clinical trial using tocilizumab (anti–IL-6R) and clazakizumab (anti–IL-6) have shown promising results for treating both acute and chronic rejection in kidney transplantation (90–95). Some pilot trials have also demonstrated the potential of IL6 blockers as safe and effective approach for desensitizing HS patients (96, 97).

The present study does have few limitations, specifically the small cohort size. Of note, HS patients had longer time on dialysis as compared to NS patients. Patients from the two groups were not matched based on the primary cause of ESRD. Cause of sensitization also varied among the HS patients. These clinical characteristics may not have equivalent impact on the immunological responses of transplant candidates. However, statistically meaningful sub-group analysis in terms of dialysis time, cause of ESRD or cause of sensitization was not feasible because of small sample size. Therefore, in future studies, these findings must be validated in a larger cohort of HS patients and compared with a matched cohort of NS patients. Moreover, IL17A and IL22 are also known to mediate type 3 immunity and disruption in their function has been associated with various infectious diseases, autoimmune disorders, and cancer. Recent single cell analysis has revealed the evidence of cellular heterogeneities and dynamic nature of canonical Th subsets in terms of expression of cytokines and transcription factors and suggest that depending on the nature of environmental stimuli, Th cell subsets can regulate each other's function resulting in heterogenous immune responses (98, 99). Future studies exploring the interplay of Type 1, 2 and 3 immunities and role of Th17 cells in this network may provide better understanding of T cell biology in HS kidney transplant recipients.

In summary, our data provide preliminary evidence of prevalence of a highly reactive immune system in HS patients, with the potential to elicit stronger alloimmune memory responses skewed towards a Th17 phenotype in sensitized patients. These findings offer a potential explanation for the poor transplant outcomes in HS patients and suggest that the assessment of pretransplant immune status has clinical relevance. Clinical evaluation of novel immunosuppressive regimens aimed at mitigating Th17 associated memory responses is essential to achieve optimized transplant outcomes in this challenging patient population.

## Data Availability

The original contributions presented in the study are included in the article/Supplementary Material, further inquiries can be directed to the corresponding author.
